# It's not black or white—on the range of vision and echolocation in echolocating bats

**DOI:** 10.3389/fphys.2013.00248

**Published:** 2013-09-11

**Authors:** Arjan Boonman, Yinon Bar-On, Noam Cvikel, Yossi Yovel

**Affiliations:** ^1^Department of Zoology, Faculty of Life sciences, Tel Aviv UniversityTel Aviv, Israel; ^2^Sagol School of Neuroscience, Tel Aviv UniversityTel Aviv, Israel

**Keywords:** yinpterochiroptera, yangochiroptera, FoxP2, swiftlet, oilbird, pteropodidae, hearing gene, eocene

## Abstract

Around 1000 species of bats in the world use echolocation to navigate, orient, and detect insect prey. Many of these bats emerge from their roost at dusk and start foraging when there is still light available. It is however unclear in what way and to which extent navigation, or even prey detection in these bats is aided by vision. Here we compare the echolocation and visual detection ranges of two such species of bats which rely on different foraging strategies (*Rhinopoma microphyllum* and *Pipistrellus kuhlii*). We find that echolocation is better than vision for detecting small insects even in intermediate light levels (1–10 lux), while vision is advantageous for monitoring far-away landscape elements in both species. We thus hypothesize that, bats constantly integrate information acquired by the two sensory modalities. We suggest that during evolution, echolocation was refined to detect increasingly small targets in conjunction with using vision. To do so, the ability to hear ultrasonic sound is a prerequisite which was readily available in small mammals, but absent in many other animal groups. The ability to exploit ultrasound to detect very small targets, such as insects, has opened up a large nocturnal niche to bats and may have spurred diversification in both echolocation and foraging tactics.

## Introduction

Echolocating bats use sonar (echolocation) to navigate in dark environments (Griffin, 1958). Other nocturnal mammals however (including most old world fruit bats) and nocturnal birds rely on other senses (such as vision, olfaction, or whisking) in similarly dark outdoor environments to orient (nearby), navigate (long-range), and forage. At first sight, vision and not echolocation seems the more apt sensory modality to invest in during evolution. Due to the hundreds of thousands parallel sensors (2D in each eye), vision conveys far more spatial information per time unit than echolocation (1D in each ear). Furthermore, bio-sonar information from natural scenes has a much lower angular resolution in comparison with visual information due to the relatively long wavelengths of sound compared to light. This also means that two “acoustic images” taken with a slight angular/positional difference will be much less correlated with each other than two consecutive visual images (Müller and Kuc, 2000). Indeed, all birds including those foraging in dim light, rely on vision when doing so (Thomas et al., 2002) and even those bird species (ca. 25 species) that have evolved bio-sonar seem to use it for orientation only and mainly in caves (Thomassen, 2005; Brinkløv et al., 2013).

Given these facts, why have most bats taken an entirely different path by opting for echolocation during their evolutionary development? Echolocation is surely advantageous over vision in extremely dark or lightless environments such as caves, but many bats customarily emerge from their roosts immediately after sunset at intermediate light levels (1–10 lux) when insect abundance peaks (Swift et al., 1985; Kon, 1989; Jones and Rydell, 1994; Rydell et al., 1996). In these bats, most feeding activity takes place during the first hours, thus many bats spend an important part of their foraging time at crepuscular light levels (>1 lux, Anthony and Kunz, 1977).

The extent to which bats rely on vision or a combination of vision and echolocation while foraging at such intermediate light levels is unknown. The eyes of echolocating bats have been shown to be adapted for nocturnal vision and are believed to impart best performance under ambient light that characterizes dusk (Bradbury and Nottebohm, 1969; Suthers and Wallis, 1970; Hope and Bhatnagar, 1979). Bats thus might rely on vision to a greater extent than commonly believed, but this must still be studied.

**Figure 1:**
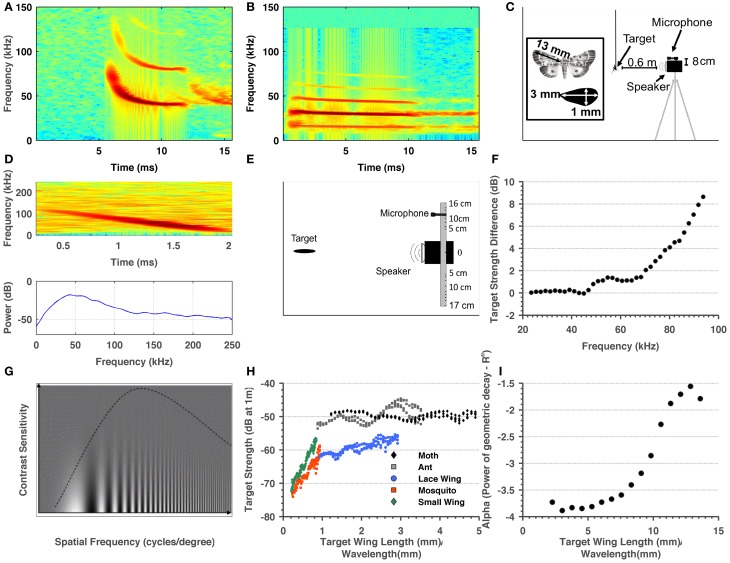
**Summary of methods and target-related parameters measured in order to estimate visual and echolocation detection ranges. (A)** Spectrogram of a typical *P. kuhlii* search call. **(B)** Spectrogram of a typical *R. microphyllum* search call. **(C)** Sketch of the ensonification setup. **(D)** Spectrogram and spectrum of a single ant echo. **(E)** Sketch of the setup used to measure the directionality of the insects. **(F)** Target strength difference between the sweep echo returning directly (microphone at 0° relative to emitter) and returning from an angle corresponding to 8 cm difference in position between center of microphone and the center of the speaker. These measurements were used to correct target strength measurements. **(G)** Adopted from Beck et al. (2007). The dependency of visual acuity on spatial frequency (X-axis) and the inverse contrast (Y-axis). It can be seen that when contrast is lower (higher on the Y-axis) acuity decreases. **(H)** Target strength estimated for four insects and one artificial object as a function of the ratio between their size and wavelength. **(I)** Alpha—the power of the (2-way) geometric attenuation as a function of the ratio between size and wavelength of the 5 cm object.

Here, we use a theoretical approach together with empirical data in an attempt to compare visual- and echolocation-based sensory performance focusing mainly on the detection range of objects provided by the two modalities. We compare two bat species that start foraging immediately after sunset, each having a different foraging strategy. We examine *Pipistrellus kuhlii* an edge space areal hawker that hunts for very small prey (e.g., mosquitos, Goiti et al., 2003) near clutter (e.g., vegetation) and *Rhinopoma microphyllum* which is an open space aerial hawker preying on large insects (mainly queen ants, Levin et al., 2009) far from clutter. *P. kuhlii* uses frequency-modulated search signals that can start as high as 95 kHz, level out at around 40 kHz, and last around 5–8 ms (Figure 1A, Kalko and Schnitzler, 1993), whereas *R. microphyllum* uses multiple harmonic search signals with a fairly constant frequency (quasi constant frequency, QCF) having the strongest harmonic at 28 kHz with a duration of 9–15 ms (Figure 1B). Both of these species (only the females in *Rhinopoma*) leave their roosts immediately after sunset when light levels are still high (>10 lux) and profit from at least an hour of hunting before darkness (<1 lux).

Our results suggest that between the two sensory modalities, vision is advantageous for the detection of large objects (e.g., cliffs, trees, etc.) and echolocation is advantageous for detecting small objects such as insects even when there still is some light. We therefore suggest that echolocation is advantageous over vision even in intermediate light levels when hunting for small prey. This finding implies a force that might have pushed the evolution of echolocation and may explain the extreme radiation and specialization found in the echolocation systems of modern bats.

## Methods

Throughout the methods whenever a parameter had to be estimated, we systematically chose parameters that overestimate the visual detection range and underestimate the echolocation detection range, motivated by the notion that if our results show any advantage of echolocation, the real advantage is probably more salient. Moreover, since we did this for several parameters, it is improbable that an error in the estimation of one single parameter would shift the general tendency we found (although it might shift the exact detection ranges).

### Ensonification and sound recording

#### Equipment

Unless stated otherwise in all experiments described below, ensonifications of various targets were performed using a ScanSpeak ultrasonic dynamic speaker (Avisoft) connected to an UltraSoundGate player116 DA converter (Avisoft). Playbacks were performed with a sampling rate of at least 500 kHz. Recordings were performed using a condenser CM16 ultrasound microphone (Avisoft). Recordings were digitized using an UltraSoundGate 116 Hm device (Avisoft) and stored onto a laptop. Sampling rate was always 500 kHz. All analysis was performed with Matlab (R2012a). Ensonifications were always performed in a sound-isolated room with acoustic foam on all walls (“the experimental room”). Frequency responses and beams of the speaker and microphones can be found in the Avisoft website: http://www.avisoft.com/

#### Target strength measurements

All ensonifications were performed in a 3 × 4 × 2.5 m^3^ acoustically isolated room with all walls (and floor) covered with acoustic foam to minimize echoes (Figure 1C). Four real insects (moth—*Noctuidae*, ant—*Camponotus*, lacewing—*Chrysopidae*, and mosquito—*Chironomidae*) along with a small wing-shaped cardboard cutout were glued to the tip of a 250 μm diameter optical fiber and hung from the ceiling at the center of the experimental room. The lengths of the insects' wings (the long axis, Figure 1C) were 18, 13, 10, 3.5 mm correspondingly. The cutout was used to estimate how much of the target's strength can be attributed to the wings. It had an elliptic shape with a long diameter of 3 mm and a short diameter of 1 mm. The speaker was mounted on a tripod at the same height as the object, 60 cm away from it with its center of beam pointing toward the object (adjusted using a laser pointer). The signal emitted was a 2 ms linear FM chirp starting from 100 kHz down to 20 kHz. The recording microphone was placed on top of the speaker (ca. 8 cm above its center, Figure 1C). The target was ensonified from different angles, thus allowing echo recordings from all around the object. Several dozen echoes were collected for each angle. The wings of the targets (or the cutout) were spread perpendicular to the direction of ensonification to ensure a good estimation of the maximum target strength of a specific object. This echo was later used for the analysis (Figure 1D, see Target Strength Data Analysis). In the visual experiments, the wings were spread similarly to ensure a comparable cross section. The incident signal was measured by placing the microphone at the target's position and recording the signal. Emission and recording gains were adjusted by a known amount to ensure maximum signal-noise-ratio (SNR, while avoiding saturation). The fiber was ensonified without an object to ensure that it did not contribute any addition to the echo. No echo could be detected from the fiber alone (in time or spectral domain). This is not surprising when taking into account that the frequency equivalent to a wavelength of 250 μm is ca. 1.4 MHz.

***Target strength data analysis***. The recorded echoes were identified by cross correlation with the emitted signal, and the four strongest echoes were used for the analysis. The following analysis was done in order to avoid any inclusion of undesired echoes or noise: First, the frequency slope of the emitted signal was measured from the spectrogram enabling estimating a time-bin for each frequency band. Next, the mean-squared spectrum of this time-bin was estimated (MSS, Matlab) and the power of the relevant frequency band was extracted from it. The same procedure was repeated for the echo and the incident signal. The difference between emission and reception could now be measured (after correcting for gain adjustments).

***Geometric decay measurements***. In previous studies it was commonly assumed that an insect can be regarded a point reflector and thus that the geometric decay of its echo is proportional to the inverse of the fourth power of its distance (1/*R*^4^). To validate this, we performed the following analysis: A 5-cm-long wing-shaped cardboard cutout (similar in shape to the one above) was glued to the optical fiber and hung from the ceiling of the experimental room. Target strength measurements (see results, Figure 1H) showed that when they are spread perpendicularly, the wings are a good approximation for the entire insect. We could not use a smaller object because of the sensitivity of our system, but since this object was larger than all of the objects we measured, if it behaves as a point reflector they would also do so. The speaker and microphone were placed as described above, but this time at increasing distances from the cutout, spanning from 50 to 100 cm. Echo analysis was performed as described above. For each frequency band the intensity decay over distance was plotted and a power function was fitted to the data (Figure 1I).

***Microphone directionality compensation***. In contrast with the expected theoretical results, target strength measurement showed a pronounced drop above 80 kHz. We hypothesized that this was an artifact resulting from the placement of the microphone 8 cm off-axis relative to the reflected echo (Figure 1C), a phenomenon that should become more pronounced in the high frequencies. To determine the extent of this effect, we repeated target strength recordings with a relatively large object (3 cm wing shaped paper cutout) placing the microphone at different azimuth angles relative to the reflection's axis (Figure 1E). This approach allowed us to estimate the effect of the angle on echo intensity across frequencies (essentially the beam of the reflected echo). We then used this estimation to correct the target-strength for the larger objects (i.e., moth, ant, and lacewing, Figure 1F). For the small-sized targets, frequencies above 80 kHz were discarded since the echoes were weak and measurements too noisy. It is important to note that this correction did not affect our detection range estimations since both bat species in the focus of this study call below 80 kHz. It only affected target strength results above 80 kHz.

#### R. microphyllum call amplitude measurements

Two wild *R. microphyllum* bats in northern Israel were caught in their roost and mounted with a 3.5 gr on-board ultrasonic microphone (Knowles, FG 23329) which recorded bats' echolocation for periods of 5 s every 30 s along one full night. Sampling rate was 94 kHz and the data was stored on an on-board flash memory. The devices were collected after several days by re-capturing the bats in their roost and the recordings were analyzed. Bats' call amplitude was determined by taking the peak or RMS voltages of the calls and converting them to dB SPL at 10 cm according to a calibrated 40 DP ultrasonic microphone (GRAS).

Calibration was performed using playbacks with the same speaker described above which were recorded by the on-board Knowles microphone and the GRAS microphone.

Because the on-board microphone was mounted on the back of the bat—it was glued using surgical glue (Permatype) between the scapula ca. 1 cm behind the head of the bat—we had to compensate for beam directionality in order to estimate the amplitude of the forward beam. Thus, a piston model (which was shown relevant for bats, e.g., Jakobsen et al., 2013) was used to estimate the difference between the peak of the main lobe and the amplitude of the call 180° behind it (Equation 1).

(1) $$R_{p}(\theta )=\left|\frac{\text{2}\cdot J_{1}\left(k\times a\times \sin(\theta )\right)}{k\times a\times \sin(\theta )}\right|$$

Where: *R*_*p*_(θ)—the ratio between pressure on-axis and at an angle θ, *J*_1_—first order Bessel function of the first kind, λ —the wavelength, *k* = 2π/λ, set to and 0.013 m, and a—the piston radius was set to 0.01 m (the bat is an oral emitter, a denotes the radius of its mouth).

This analysis resulted in a ca. −30 dB decrease at 150°. The piston model is symmetric thus having a peak equal to the main one at 180° which is not the case for the bat. Since we wanted to be sure not to overestimate echolocation detection range we used a safer –20 dB compensation value thus probably underestimating echolocation.

#### P. kuhlii call amplitude measurements

Wild bats were recorded in a park in Tel-Aviv using a 12 synchronized microphone array (USG1216H 12 channel A/D converter, Avisoft, Knowles microphones FG23329). The array was arranged with 10 microphones in a straight line (equally spread over 1.5 m at a height of 1.5 m above ground), and two additional microphones on a vertical axis, one 27 cm below and one on the ground 1.5 below the central horizontal microphone.

The recordings from 4 of those 12 microphones—the leftmost, middle, rightmost, and lowest ones—were later used to estimate the bat's position and thus reconstruct its flight trajectory. This was done by an in-house code (Matlab), which implemented a Time Difference of Arrival (TDOA) algorithm. This made it possible to estimate the distance of the call's origin from the microphones. Only calls that were part of a flight path heading toward the array (i.e., with their horizontal peak falling within the array) were analyzed. We could not tell if the bat was pointing its beam above the array. Actually this was probably the case because bats were flying above the array so our SPL estimations were therefore probably underestimations of the real emission levels.

The call's amplitude in dB SPL (peak and RMS) was then derived using a calibrated microphone (GRAS, 40 DP) which was calibrated relative to the array's microphones. Geometric attenuation was compensated for, assuming a 6 dB decay for every doubling of the distance. Atmospheric attenuation was accounted for with alpha = 0.3 m^−1^ (according to a temperature of 30°C and a humidity of 70%, taken from a table). Ambient light levels were recorded at the same time (see below).

#### Maximal echolocation detection range calculation

The maximal echolocation detection range was calculated by numerically solving the RADAR/SONAR equation (Skolnik, 1970) for the distance variable *R*.

(2) $${\text{P}}_{r}=\frac{P_{t}\cdot \sigma_{bs}\cdot e^{-2\alpha (R-0.1)}}{{\left(\frac{R}{0.1}\right)}^{4}}$$

Where *P*_*r*_ is the power returning back to the bat's ear (per m^2^, see below), *P*_*t*_ is the power transmitted by the bat, σ_*bs*_ is the backscattering cross-section, α is the atmospheric attenuation [alphas were 0.1 m^−1^ for *Rhinopoma* (28 kHz) and 0.3 m^−1^ for *Pipistrellus* (40 kHz) according to a temperature of 30° and a humidity of 70%] and *R* is the distance of the object from the bat.

The target's cross-section was calculated from the target strength by this formula:

(3) $$TS=10\text{log}\left(\frac{\sigma_{bs}}{4\pi r^{2}}\right)$$

Where *r* is the distance from the target. In our case, the target strength was calculated at a distance of 60 cm, so *r* was set to this value. The transmitted power used was the maximal call strength measured in the abovementioned experiments, in dB SPL at 10 cm.

Following the debate in the literature about the hearing threshold of bats (Moss and Schnitzler, 1995), two alternative simulations representing the two extreme hypotheses were performed, one with the minimum *P*_*r*_ set to 0 dB (see for instance Kick, 1982), and the other with it set to 20 dB (see for instance Griffin et al., 1960). *P*_*r*_ essentially takes into account the brain's hearing sensitivity but also the ear's gain (or area) and is actually in units of W/m^2^. In our opinion the 0 dB threshold is more suitable for our analysis because it represents the maximum hypothetical threshold bats exhibit in the lab while the higher threshold (20 dB) represents the actual sensitivity observed in the field (when noise in present). Since in the visual estimation (see below), we use the maximal hypothetical range estimated in the lab with no noise, the fair comparison would be the 0 dB threshold. Still, we show both results.

#### Visual experiments

The following measurements (Light Measurements, Contrast Measurements, Reflectivity Measurements) were necessary prerequisites for estimating visual detection range according to the methods which will be described below (Maximal Visual Detection Range Calculation).

***Light measurements***. Ambient light illuminance levels in the various experiments were captured by a Fourier Education MultiLogPRO data logger with a 0–300 lux light detector. The accuracy of the sensor is ±4% (thus ca. 0.04 lux for the range we were measuring). We define the range between 1 and 10 lux as intermediate light level. This ambient light is typical for the time of the day between dusk and complete darkness when many bats are active and many insects are available. We define darkness as ambient luminance <1 lux.

***Contrast measurements***. The four targets mentioned above were photographed at the same light conditions against two different backgrounds: sky and vegetation. The photos were taken by a Canon EOS Kiss X5 camera set without flash. Pictures were taken from around the time bats emerge from their roosts until darkness (i.e., 1–10 lux).

The Weber contrast is essential for calculating the detection range in our first method. It represents the contrast between the object and the background and was calculated by measuring the average pixel amplitude of the target and of the background (only for the red sensors).

(4) $$\text{Weber contrast}=\frac{I-I_{b}}{I_{b}}$$

Where *I* is the intensity of the object and *I*_*b*_ is the intensity of the background (i.e., sky or vegetation). We only used the higher contrasts (e.g., with the sky background) thus overestimating visual detection range. We discuss the effect of lower contrast in the discussion.

***Reflectivity measurements***. Target reflectivity is the proportion of the photons that hit the target returning from it. It was used in the second approach for calculating the visual detection range. To measure target reflectivity the targets described above were taken to a dark room in which the walls are black assuring minimal light reflectance, and hung from the ceiling attached to the optical fiber. They were photographed by a Canon EOS Kiss X5 camera set to a 1/60″ exposure time and an aperture of *f*/4, with a constant flash burst. The reflectivity was calculated by comparing the target's pixel intensity to that of a white board (100%) while making sure that the white is not bleached (stayed under the saturation level of the camera-sensor).

(5) $$\text{reflectivity}=\frac{I}{I_{b}}$$

Where *I* is the intensity of the object and *I*_*b*_ is the intensity of the white paper. The values calculated were: Moth—0.6, Ant—0.3, Lacewing—0.55, and mosquito—0.45.

***Maximal visual detection range calculation***. Two different approaches were used to estimate the maximal visual detection range for the experiment targets. The first is based on the visual acuity measurements which are a measure of the minimum resolution angle found in previous studies (see Table 2 in Eklöf, 2003, for a summary). Because visual acuity measures the maximal resolution range, and we were interested in the maximal detection range (which might be longer), we had to find a way to translate visual acuity into a detection threshold (or sensitivity).

We relied on the results of Lie (1980) who showed that in the far periphery of the human eye (where photo-receptor composition includes rods-only and should be most similar to the bat's eye) the minimum detection angle is ca. 3.5 times smaller than the minimum resolution angle for contrast levels similar to the ones found in our study. We compared Lie's measurements in the photopic or the scotopic regimes and both generated similar results. For *P. kuhlii* we used an acuity angle (0.8°) smaller than that found for the species that are phylogenetically closest to ours (0.9°, *P. rueppellii* and *P. nanus*, Table 2 in Eklöf, 2003) and the same as the smallest angle measured for any vespertilionid (Suthers and Wallis, 1970). Since there was no estimation for a *Rhinopomatidae* bat, for *R. microphyllum* we took a value that is close to the smallest value found for any bat—0.5° (Table 2 in Eklöf, 2003, e.g., Suthers, 1966; Chase, 1972). It should be emphasized that bat acuity measurements found in the literature for micro-bats vary a lot ranging between 0.3 and 5° (Altringham and Fenton, 2005) and we chose values that are very close to the lower bound to ensure overestimation of the visual detection range. The maximal detection range was then derived following basic geometry:

(6) $$D=\frac{S}{2\cdot \tan\left(\frac{V}{2\cdot 3.5}\right)}$$

Where *D* is the detection range, *S* is the target's longest dimension, and V is the minimum acuity angle converted into radians. 3.5 is the factor taken from Lie (1980).

It is important to note that the visual acuities of the species that we used (i.e., 0.8 and 0.5°) were estimated for stimuli with much higher contrast than any of our targets (white and black stripes, e.g., Bell and Fenton, 1986), and therefore this compensation of 3.5 is likely an overestimation (see Figure 1G to see how acuity depends on contrast). Moreover, Hecht and Mintz (1939) actually showed that visual acuity and visual sensitivity are virtually the same (in humans) as light intensity approaches threshold. In fact, the only study that tested visual range (or sensitivity, Bell and Fenton, 1986) found a value of 1° for *Eptesicus fuscus* which is very similar to *P. kuhlii* in both its echolocation signal and foraging style. This implies a 4 time over estimation in our study (0.8/3.5 = 0.22°).

Because the visual measurements above were based on several assumptions, we used a second different approach to validate our estimations. This approach was to directly estimate the photon flux necessary for object detection by a bat. This approach can be thought of as equivalent to estimating the minimal sound pressure level required for sound detection. Here, we relied on the results of Ellins and Masterson (1974) that tested the big brown bat's (*E. fuscus*) discrimination performance of a white vs. a black card under different light conditions.

The photon flux (photons per unit area per second) of a reflecting object at a distance *D* can be estimated from to the ambient illuminance *E* (light power per area—lux), the reflectance of the object ρ (measured in percent, %), and the object's area, *A* (m^2^), according to the following proportion (Ryer's, 1997):

(7) $$\text{Flux Intensity}\propto \frac{E\cdot \rho \cdot A}{\pi \cdot D^{2}}$$

Note that flux depends on the available photons (*E*) and the object's “visual target strength” (ρ · *A*) and decays according to geometric spreading (1/*D*^2^). Ellins and Masterson (1974) found that bats perform at chance level, and thus could not detect the object anymore for a white object positioned at a distance of *D* = 0.37 m with an area of *A* = 51.6 cm^2^, a reflectance of ρ = 89.5%, at a light level between 0 and 0.00079 lux (we thus used the middle *E* = 0.000395 lux).

When plugging these numbers in equation 5 one reaches a threshold of 4.24e-6 lux/steradian. This is thus an estimation of the minimal photon flux necessary for detection of an object by the big brown bat. The visual acuity reported for this species is 0.7–1° thus similar to the acuities we used above (Bell and Fenton, 1986; Koay et al., 1998). We could now use this threshold along with the reflectivity measurements of the objects in our experiment (ρ, see above) and the targets' surface area (A, measured with an image processing tool—imageJ) to estimate *D* (Equation 7)—the maximum detection range for the objects in this study under different ambient light levels (5 or 10 lux). A was estimated with the insect wings spread perpendicular to the camera, thus in a posture comparable to the ensonification posture. Notice that our estimations thus assume that detection range increases linearly with illuminance which is very likely an overestimation.

## Results

### Do bats use echolocation under intermediate light levels?

**Figure 2:**
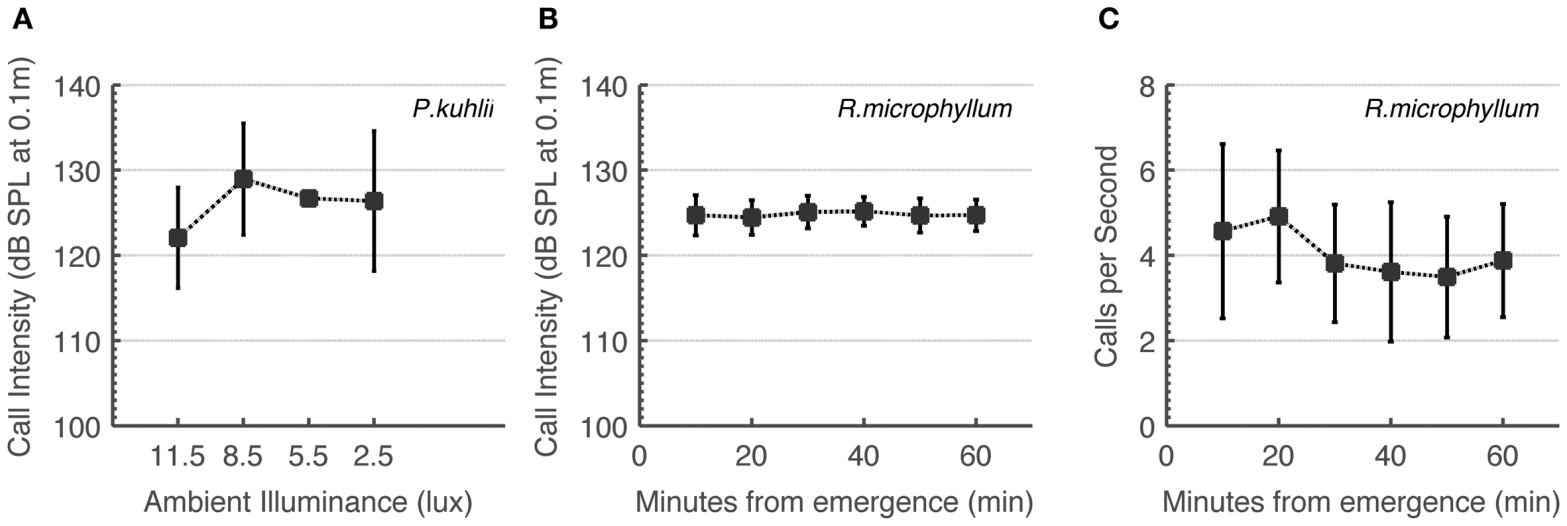
***R. microphyllum* and *P. kuhlii* use echolocation under intermediate light levels to the same extent as in the dark. (A)** *P. kuhlii* call intensity as a function of ambient light levels. **(B)** *R. microphyllum* call intensity as a function of time after sunset. **(C)** *R. microphyllum* call rate as a function of time after sunset. Both **(B,C)** were measured at the beginning of the month so that moonlight was limited. All panels show means and standard deviations.

Some studies have implied that bats “turn-off” echolocation when light is sufficient to use vision (e.g., Bell, 1985). We therefore first had to prove that the bat species in the focus of this study use echolocation under intermediate light levels. To do this, we monitored changes in calling rate and calling intensity. On-board recordings of *Rhinopoma* during the first hour after sunset show that these bats do not increase call intensity or call rate as light levels decrease (Figures 2B,C). Statistical analysis actually showed a significant decrease in calling rate (One-Way ANOVA for each bat, *F*_5_ > 11, *P* < 10^−9^), but we believe this to be a result of bats flying with fewer conspecifics as distance from the roost increases. In the *Pipistrellus* bat we could not quantify call rate, but we can report that all catching maneuvers observed by us were accompanied by feeding buzzes independently of ambient light levels. We found significant changes in call intensity which nevertheless did not reveal any systematic increase or decrease over time in *Rhinopoma*, and no significant changes in *Pipistrellus* as light levels decreased (One-Way ANOVA for each *Rhinopoma*, *F*_5_ < 2.5, *P* < 0.05 and One-Way ANOVA for all *Pipistrellus* bats, *F*_3_ < 2, *P* > 0.05, Figures 2A,B). These results suggest that echolocation is used by these bats irrespective of ambient light levels as long as they are below 10 lux.

### Echolocation detection range

It is very hard to estimate the exact detection range for a small object (e.g., an insect). The RADAR/SONAR equation (Equation 2) is usually used for this purpose, but two of its important parameters, the hearing sensitivity of the bat and the target strength of the object, are difficult to measure. Moreover, one can measure the bat's signal intensity (peak or RMS) but this signal is usually composed of many frequencies while it is not clear how to model the brain's temporal-spectral integration for such a signal. To estimate detection ranges we measured the target strength of five objects with different sizes, we measured bats' emission intensity and estimated the geometric attenuation factor. Bats' emission intensity corresponded with estimations for other species varying around peak levels of 130 dB SPL (Holderied and Von Helversen, 2003; Surlykke and Kalko, 2008). Our target strength measurements (Figure 1H) confirm previous findings (Waters et al., 1995; Houston et al., 2004). The measurements also showed the expected relation between target strength and the ratio between the size of the target and the wavelength, i.e., a steady increase for ratios smaller than 1 and saturation thereafter.

Detection ranges were estimated for two hearing thresholds (0 and 20 dB SPL, Tables 1,2) and ranged between 2 and 3.5 m in *Pipistrellus kuhlii* and 2 and 6.5 m for Rhinopoma microphyllum for the higher threshold, and between 4 and 7 m (*P. kuhlii*) 5.5 and 14 m (*R. microphyllum*) for the lower threshold when using peak emission levels. Interestingly, our data suggests that *R. microphyllum* performs better for all objects due to its lower emission frequency which suffers from less atmospheric attenuation. This is true even for the smallest objects for which the higher frequency of *P. kuhlii* results in increased target strength. The artificial wing-like cutout had a target strength (and thus a detection range) which was almost identical to that of the similar sized mosquito, confirming that for such small insects when the wings are spread perpendicular to the axis of ensonification they are the main echo source.

**Table 1:** **Echolocation and visual detection range for *P. kuhlii***.

**Object/method**	**Acoustic (m)**	**Acoustic (m)**	**Visual method 1(m)**	**Visual method 2(m)**	**Visual method 2(m)**
	**(20 dB)**	**(0 dB)**		**10 lux**	**5 lux**
Moth (18 mm)	4 (3)	7 (5.5)	4.5	6	4
Ant (13 mm)	3.5 (2.5)	7 (5.5)	3.5	3.5	2.5
Lace wing (10 mm)	2.5 (1.5)	5 (4)	2.5	1.5	1
Mosquito (3.5 mm)	2 (1)	4 (3)	1	0.5	0.5
Artificial wing (3 mm)	2 (1)	4 (3)	–	–	–

Echolocation-based detection ranges are shown for four types of prey and one artificial small object. Ranges are shown for two alternative hearing sensitivities (0 or 20 dB SPL) and for either the peak or RMS (in brackets) emission levels. Visual detection range is presented for two different methods, (1) based on visual acuity and (2) based on photon flux. The second method is estimated for two different light levels (5, 10 lux). All ranges are given in meters.

**Table 2:** **Echolocation and visual detection ranges for *R. microphyllum***.

**Object/method**	**Acoustic (m) (20 dB)**	**Acoustic (m) (0 dB)**	**Visual method 1(m)**	**Visual method 2(m) 10 lux**	**Visual method 2(m) 5 lux**
Moth (18 mm)	6.5 (4.5)	14 (10.5)	7	6	4
Ant (13 mm)	5.5 (3.5)	12.5 (9)	5	3.5	2.5
Lace wing (10 mm)	3 (2)	8 (6)	4	1.5	1
Mosquito (3.5 mm)	2 (1.5)	5.5 (4)	1.5	0.5	0.5
Artificial wing (3 mm)	2 (1.5)	5.5 (4)	–	–	–

All ranges are in meters. See Table 1 for details.

The maximal detection range for small targets while assuming a hearing threshold of 20 dB corresponded well to reaction distances of bats to prey that have been measured for hunting Pipistrelle bats in the field (1–2 m, Kalko and Schnitzler, 1993) while the 0 dB estimations corresponded with detection ranges estimated for *E. fuscus* in the lab (3 m for a 5 mm sphere, Kick, 1982).

A recent paper has proposed to model insect wings as planar reflectors instead of point reflectors to calculate the target strength of insects (Armstrong and Kerry, 2011). We empirically tested these calculations for a large (5 cm long) wing-shaped cutout (see methods) and found that even the largest wing-surfaces bats encounter still behave much more than a point reflector than like a planar reflector (Figure 1I).

### Visual detection range

The exact visual detection range for a small object is a complicated function which depends on the contrast, the spatial frequencies of the object and the transfer functions of the eye. Very little research has tried to assess the behavioral or physiological visual detection range of bats and moreover, the physiology of the bat eye is far from being understood (see Eklöf, 2003, for a summary). We therefore used two alternative approaches to estimate the range from which the bats studied here can detect four real insects.

In the first approach, we used the visual acuity (or maximum resolution) which represents the minimum separable angle for two nearby objects and which was estimated for several bats (e.g., Bell and Fenton, 1986; Eklöf, 2003). We translated visual acuity into detection range (see methods). In the second approach we relied on behavioral experiments performed in *E. fuscus* (Ellins and Masterson, 1974) and tried to estimate the minimum photon flux a bat can detect. Importantly, both methods provided similar ranges, strengthening our confidence in the estimations. Estimations ranged between 0.5 and 7 m depending on object size and were consistently lower than the equivalent echolocation based detection range (Tables 1, 2). Notice that the second method gives different estimations depending on the illuminance.

### Comparing vision with echolocation

In the analysis above we systematically chose parameters that overestimate visual detection range and underestimate echolocation detection range. This was to ensure that any advantage found for echolocation is real and might even be more salient in reality. In brief (see methods for full details), the decisions taken to overestimate vision include: (1) using the higher contrast among the two measured (sky vs. vegetation). (2) The assumption that sensitivity is 3.5 higher than acuity. (3) Using the smallest visual acuity measurements reported in the literature. (4) In the second approach—assuming that range increases linearly with illumination. In echolocation we probably underestimated the emitted sound pressure level (by several dB at least).

**Figure 3:**
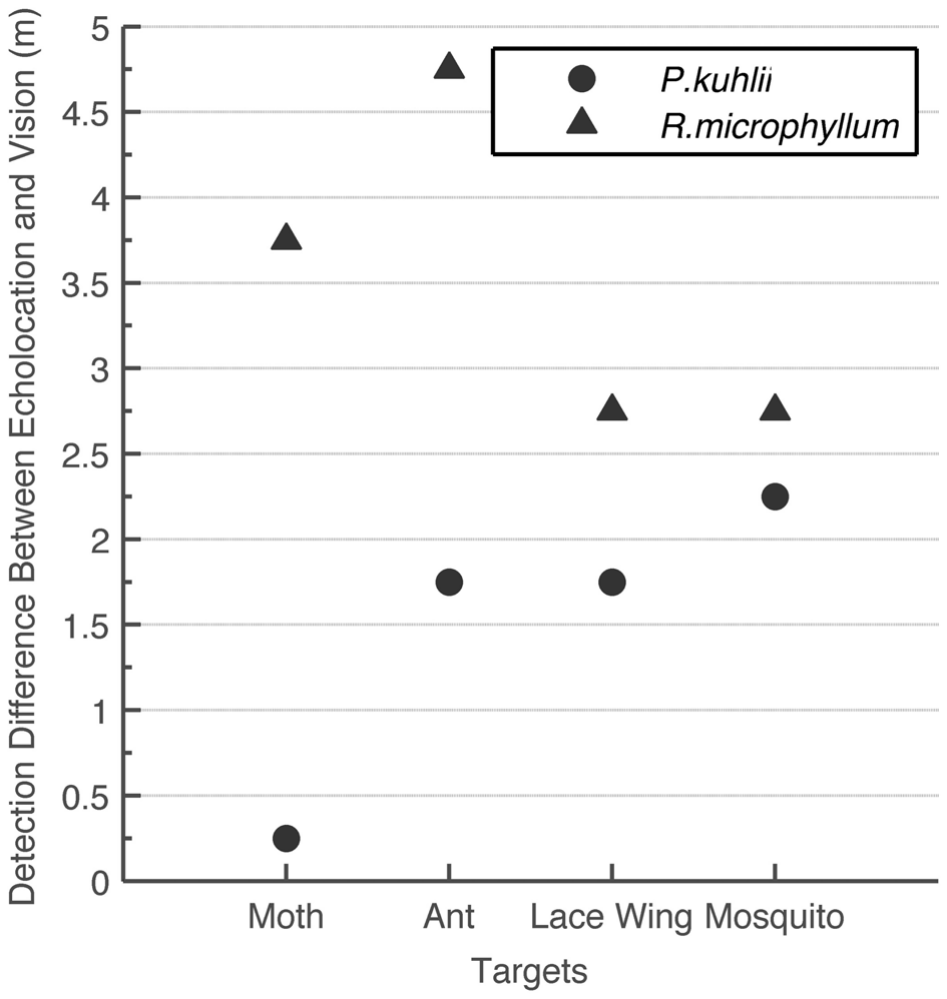
**Comparison between visual and echolocation detection ranges.** The difference (in meters) between echolocation detection range and the visual detection range for two bat species and four insects. In all cases echolocation detection range was higher than visual detection range.

Despite using this conservative approach, we found that for detecting small objects, echolocation is advantageous over vision under the light conditions examined (intermediate to low light levels; Figure 3). Statistical analysis confirms that the differences between echolocation and visual detection ranges are significant for both species (*t*-test, *P* < 0.05 for *P. kuhlii* and *P* < 0.01 for *R. microphyllum*). When comparing the two modalities statistically, we used the average of the two visual estimations (using the 10 lux condition for the second approach) and averaging the two peak acoustic estimations (acquired for two hearing sensitivities). For each species, we then subtracted the echolocation range from the visual range and ran a *t*-test to check that the difference is significantly higher than zero. Moreover, even if we were to use the 20 dB worse estimation (which we find unsuitable, see methods) vision would become slightly beneficial over echolocation (0.5 m) only for one case of detecting a moth by *P. kuhlii*.

## Discussion

### Echolocation is advantageous for finding small prey

Apart from being a superior navigational sense under extremely dark circumstances (e.g., Griffin, 1958), we find that echolocation is also superior to vision for detecting and tracking small insects even at intermediate light levels (1–10 lux). This seems to be the case for both species we examined even though they use different signal designs and hunt for different sized prey. Figure 3 even suggests that the “sonar advantage” is most pronounced at the typical insect size each bat species eats: flying ants (*Rhinopoma*), mosquitos (*Pipistrellus*). Estimating the (visual or echolocation) detection range requires several assumptions. In our analysis however, we systematically made assumptions that *over*estimate visual detection range and *under*estimate echolocation detection range. This guarantees that the advantage we found for echolocation is likely to be real. The advantage of echolocation over vision has in fact been suggested once before (Fenton et al., 1998; Altringham and Fenton, 2005) suggesting that for a 19 mm sphere echolocation detection range is five times larger than the visual range in dim light (exact light levels not stated). The fact that we find a smaller advantage (up to 2 folds) is probably a result of our conservative approach.

Echolocation provides several additional advantages over vision. One such advantage is that it tends to provide more continuous tracking, losing the object only when it disappears behind a background. In vision on the other hand, even if the target stays in front of any object its contrast might change dramatically depending on the background, causing it to disappear frequently. We found that a vegetation background, as opposed to the sky led to a 3–5 fold decrease in contrast (Table 3) which would result in a 3–5 fold decrease in the visual detection ranges reported above.

**Table 3:** **Insect contrast against different backgrounds and under different light levels**.

**Object/background**	**Sky**		**Ground/vegetation**	
	**10 lux**	**0.5 lux**	**10 lux**	**0.5 lux**
Moth	0.93	0.93	0.33	0.31
Ant	0.94	0.91	0.46	0.37
Lace wing	0.82	0.70	0.33	0.12
Mosquito	0.75	0.66	0.24	0.08

In addition, echolocation also provides much more accurate estimations of the distance of an object, its velocity (calculated by integrating several echoes) and sometimes even the distance of the background behind it (Aytekin et al., 2010; Melcón et al., 2011).

Despite these advantages of echolocation over vision, we cannot rule out the possibility that in some species or in some situations (especially when contrast is high) visual cues could assist in prey detection (e.g., Bell and Fenton, 1986; Eklöf et al., 2002). Vision has some advantages such as not suffering from sensory interference that might arise when conspecifics forage together while using similar frequencies (Ulanovsky et al., 2004; Chiu et al., 2008; Bates et al., 2010). We conclude that much more behavioral and physiological research is necessary to understand the extent to which echolocating bats rely on vision.

### Echolocation and visual detections range for large objects

Large landscape objects such as forest edges have recently been estimated to have a maximal echolocation detection distance by bats of about 50 m (Stilz and Schnitzler, 2012). Other studies have estimated even longer ranges (e.g., 90 m in Holderied and Von Helversen, 2003) but the order of magnitude is similar. The main reason for this limited distance is the strong atmospheric attenuation of ultrasound. The visual detection range for large objects is undoubtedly several orders of magnitude larger because sound attenuates much faster than light (e.g., Altringham and Fenton, 2005). For instance, when using visual acuity estimations with an acuity angle of 0.5°, a detection range of 2 km is reached for a sphere of 5 m diameter.

### Echolocating bats integrate visual and sonar based information to perceive the world

**Figure 4:**
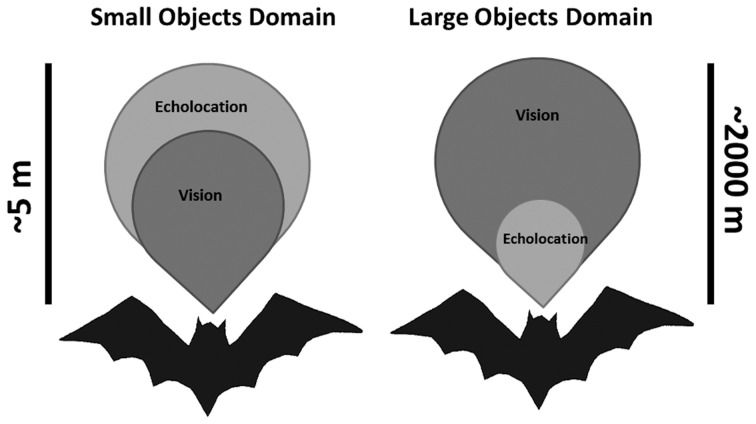
**Sketch depicting the two sensory domains used by echolocating bats that are flying in intermediate light levels.** Left—echolocation is slightly advantageous when searching for small prey. Right—Vision is strongly advantageous when avoiding large obstacles. Scale bars depict the approximated detection distances, but are qualitative and not quantitative (especially for the large objects domain).

We find that both bat species tested here rely on echolocation even when light levels are high enough to allow good vision. Since the detection range of even large objects using echolocation is short (no more than 100 m, Holderied and Von Helversen, 2003; Stilz and Schnitzler, 2012) we hypothesize that in intermediate light levels characteristic of dusk, many bats use bimodal sensing. On the one hand, bats predominantly rely on vision for orientation, navigation and avoiding large background obstacles (e.g., Williams and Williams, 1967; Chase, 1981; Mistry, 1990), while on the other hand they mainly rely on echolocation when searching for small prey (Figure 4). Clearly, these two are not mutually exclusive behaviors. A *P. kuhlii* bat which uses echolocation to search for insects probably uses vision at the same time to keep track of nearby background targets such as trees and buildings. A *R. microphyllum* bat will search for queen ants in open space using echolocation while visually following the distant terrain to monitor its location relative to the roost. The brains of these two bats must therefore constantly integrate two streams of information acquired by two different modalities into a single image of the world.

### The evolution of echolocation

Many previous discussions on the evolution of echolocation in bats have focused on whether echolocation or flight evolved first (Speakman, 2001; Denzinger et al., 2004; Simmons et al., 2008). One important question that has not been sufficiently addressed in our opinion is how echolocation could evolve from a rudimentary- (as in echolocating birds) and probably complementary sensory system into the highly complex sonar system observed in bats today. Our results show that echolocation improves the ability of bats to detect small objects even when there is sufficient light for using vision to orient and avoid large obstacles. If we follow the evolutionary scenario proposed by Simmons and Geisler (1998) of flying bats first using vision only (Simmons et al., 2008), echolocation could improve gradually for the detection of increasingly small targets in parallel to using vision for orientation and navigation. In fact, the selective advantage of evolving echolocation is still given, even if the detection range it allows is similar to vision (and not better than vision). This is because the integration of multiple sensory information leads to a more robust percept (Deneve and Pouget, 2004).

In this evolutionary discussion we focus on the gains of certain sensory abilities and not on their costs. We hope that future studies can shed light on the additional maintainance costs of evolving specialized nocturnal eyes in comparison to the additional costs of emitting frequently in ultrasound (Speakman and Racey, 1991).

Our finding that the ability to detect insect-like (small-) targets is the main advantage of echolocation raises two interesting questions: How advantageous is the detection of small targets in terms of food intake, and if it is advantageous why didn't echolocation evolve for insect detection in the other group of flying vertebrates–birds?

### Advantages of small target detection in terms of food intake

Several studies have shown that in aquatic-, or water rich habitats including desert stream habitats chironomids (mosquitos) make up 53–94% of the emerging aquatic biomass, with 90% of them being insects less than 7 mm length (Jackson and Fisher, 1986; Gray, 1993; King and Wrubleski, 1998; Lynch et al., 2002). Many of these insects have a peak of activity around dusk when many bats start foraging (Racey and Swift, 1985; Rydell et al., 1996). Furthermore, there are many chironomid species, some of which are active even during the winter months of harsh continental zones (Krasheninnikov, 2012) so that the availability of Chironomidae as prey is nearly all year round. This is in contrast to moths whose seasonal occurrence is very peaked (Yela and Herrera, 1993). Bats in temperate zones, do predominantly feed on small Diptera (Vaughan, 1997; Dietz et al., 2007) which can be as small as 3 mm wing-length (Houston et al., 2004) and a recent molecular diet analysis of two African molossids also showed diets to be largely composed of dipteran prey (Bohmann et al., 2011). It seems therefore that the ability of bats to detect small prey in intermediate light levels has opened up for them a new and significant niche.

### Why didn't birds evolve echolocation for insect detection?

Our data show that the use of high frequencies (ultrasound) is essential for the detection of small targets (Figure 1H). Ultrasonic hearing is common in mammals even among non-echolocating mammals such as tarsiers (Ramsier et al., 2012), tree shrews (Heffner et al., 1969), rats, and mice (Heffner and Heffner, 1985), whereas in birds ultrasonic hearing has probably never evolved (Necker, 2000). Manley (2012) details the essential evolutionary steps mammals went through to obtain ultrasonic hearing: about 230 million years ago a middle ear consisting of three ossicles instead of one, and 100 million years later a tuned basilar membrane, specialized prestines and a coiled cochlea. Since birds only had one ossicle at their disposal and lacked the other adaptations, evolution to receive ultrasound was less probable and has not (yet) evolved. Another reason why ultrasonic hearing did not evolve in birds might be that since their hearing canals are coupled even birds with small heads can estimate the direction of a sound source with high precision. In small mammals however, since the ears are uncoupled, only in high frequencies would wavelengths be small enough to allow precise directional hearing (Heffner and Heffner, 2008; Christensen-Dalsgaard, 2011). This ability to hear and locate the rustling (highly ultrasonic) noises of an approaching predator would provide a selective advantage to small mammals and thus would be probably passed on quickly.

The inability of birds to operate in ultrasound has not prevented them from using audible echolocation (probably 25 species; Brinkløv et al., 2013), nor from being nocturnal. We hypothesize that the lack of ultrasound reception and hence the ability to detect small (insect-) targets has kept birds out of the niche of insectivorous bats. Of the 10,000 bird species inhabiting our planet none are likely to be able to detect small targets (Griffin and Suthers, 1970; Griffin and Thompson, 1982) by using echolocation, whereas more than 1000 species of bats are. Of the purely visually orienting birds there are only about 80 species of birds (nightjars) which exclusively feed on insects at night and these are limited in the following ways: (1) Dietary studies suggest that nightjars rely on catching large (13 × 6 mm) insects (mainly Coleoptera/Lepidoptera, very few Diptera) for their survival (Taylor and Jackson, 2003). (2) They are active in twilight rather than at night and other than in bats they require a minimum light level of 0.03 mW/m^2 ~1/30 lux to be active (Jetz et al., 2003). They usually forage by perching on the ground and detecting insects against the sky. In nightjars we do not (or only rarely) see specializations such as trawling, gleaning, or foraging in extreme clutter or extreme open space (Holyoak, 2001).

At the same time echolocation has allowed bats to specialize on alternative detection modes, such as flutter detection using Doppler shifts (Schnitzler, 1970), or gleaning prey from vegetation (Neuweiler and Fenton, 1988), which, in turn, might have pushed their radiation into different climatic zones on earth and into many different niches.

In conclusion, we hypothesize that the ability to hear ultrasound has provided mammals with the unique potential to detect small prey items by means of sonar. Bats have probably exploited this potential to an extreme degree and have capitalized on the vast biomass of small flying insects active around dusk. Here, we bring strong evidence that they could use echolocation and vision in a complimentary fashion which would enable a gradual evolution of echolocation.

### Conflict of interest statement

The authors declare that the research was conducted in the absence of any commercial or financial relationships that could be construed as a potential conflict of interest.
